# Causal Association Between Thyroid Function and Myeloproliferative Disease: A Two‐Sample Mendelian Randomization Study

**DOI:** 10.1155/ije/6688740

**Published:** 2026-06-27

**Authors:** Jingjing Xiang, Jun Yan, Jianping Shen, Mengke Mao, Zhiyin Zheng, Shu Deng

**Affiliations:** ^1^ Department of Hematology, The First Affiliated Hospital of Zhejiang Chinese Medical University (Zhejiang Provincial Hospital of Chinese Medicine), Hangzhou, Zhejiang, China, zjhtcm.com; ^2^ Laboratory of Chemistry and Physics, Hangzhou Center for Disease Control and Prevention (Hangzhou Health Supervision Institution), Hangzhou, Zhejiang, China

**Keywords:** chronic myeloproliferative disease, hyperthyroidism, Mendelian randomization, myeloproliferative disease, thyroid function

## Abstract

**Background:**

Thyroid dysfunction has been linked to hematologic abnormalities, but whether thyroid function has a causal role in myeloproliferative diseases (MDs), including chronic myeloproliferative disease (CMD), remains unclear. We used Mendelian randomization (MR) to assess the potential causal effects of thyroid‐related traits on MD risk.

**Methods:**

A two‐sample MR analysis was performed using genome‐wide association study (GWAS) summary statistics. Outcome data for CMD and MD, excluding chronic myelogenous leukemia (CML), were obtained from the Finnish R12 cohort (European ancestry). Genetic instruments for thyroid traits—including thyrotropin (TSH), free thyroxine (FT4), free/total triiodothyronine (FT3/TT3), FT3/FT4 and TT3/FT4 ratios, high/low TSH, and genetic liability to hyperthyroidism and hypothyroidism—were selected at genome‐wide significance (*p* < 5 × 10^−8^), clumped for linkage disequilibrium (*R* ^2^ < 0.001, 10,000 kb), and required F‐statistics > 10. Primary causal estimates were obtained using inverse‐variance weighted (IVW) MR, with sensitivity analyses using MR‐Egger, weighted median, weighted mode, MR‐PRESSO, heterogeneity tests, and leave‐one‐out analyses. Steiger tests were applied to assess directionality, and false discovery rate (FDR) correction was used for multiple comparisons.

**Results:**

Most thyroid function measures (TSH, FT4, FT3, TT3, hormone ratios, and hypothyroidism) showed no evidence of a causal association with CMD or MD excluding CML (IVW *p* > 0.05 *p* > 0.05 *p* > 0.05). A nominal association was observed between self‐reported hyperthyroidism/thyrotoxicosis and CMD, but the effect estimate was implausibly extreme (IVW OR = 5.53 × 10^−14^, 95% CI: 7.78 × 10^−27^–0.3935, *p* = 0.0432) and did not remain significant after FDR correction. After outlier removal, genetically predicted hyperthyroidism was nominally associated with MD excluding CML (OR = 0.8806, 95% CI: 0.7954–0.9750; *p* = 0.0144), but this also did not survive FDR correction (FDR = 0.2015). Sensitivity analyses and pleiotropy tests did not indicate strong directional pleiotropy for the nominal signals, and Steiger tests supported the hypothesized exposure‐to‐outcome direction.

**Conclusions:**

In this MR study, we found no robust evidence for a causal association between most genetically predicted thyroid function traits and MD after multiple‐testing correction. The nominal signals observed for hyperthyroidism were phenotype‐dependent and statistically unstable, particularly for self‐reported CMD. These results emphasize that previously observed associations may be driven by nongenetic factors or clinical confounding.

Jingjing Xiang and Jun Yan were co‐first authors.

## 1. Introduction

Myeloproliferative diseases (MDs) represent a group of chronic hematologic malignancies characterized by the clonal proliferation and overproduction of one or more mature blood cell lines. Clinically significant subtypes, including essential thrombocythemia (ET), polycythemia vera (PV), and primary myelofibrosis (PMF), carry notable population burdens, with estimated annual incidence rates of approximately 1.03, 0.84, and 0.47 per 100,000 individuals, respectively [1]. While clinical presentation varies, ranging from asymptomatic discovery to significant symptoms like fatigue, splenomegaly, or thrombosis [2], a major challenge in MD management lies in mitigating the substantial risks of disease progression, transformation to acute leukemia, and life‐threatening vascular events [3, 4]. Current therapeutic options provide benefit but are not curative and have limitations, highlighting an urgent clinical need to identify novel, potentially modifiable risk factors that could inform more effective prevention or targeted intervention strategies.

Thyroid function, primarily assessed through markers like thyroid‐stimulating hormone (TSH) and free thyroxine (FT4), plays a key role in regulating metabolism and cellular processes throughout the body [5]. Interestingly, clinical observations have suggested a potential interplay between thyroid dysfunction and hematologic disorders, including MD. For example, Wang et al. [6] noted increased basal metabolism in patients with PV, and Modello et al. [7] found a lower prevalence of thyroid enlargement and nodules in patients with hematologic diseases compared to controls. In contrast, Aggarwal et al. [8] reported that thyroid dysfunction was more prevalent in patients with thrombocythemia. However, biological mechanisms involving thyroid hormone effects on hematopoietic stem cell function and the bone marrow microenvironment offer potential explanations. These contradictory findings highlight the uncertainty regarding whether thyroid dysfunction contributes to MD development or progression. Additionally, observational studies are often limited by confounding factors, such as environmental influences and immune‐related comorbidities, making it difficult to establish a definitive causal relationship.

Mendelian randomization (MR) provides a robust statistical approach to probe causal relationships by using genetic variants associated with an exposure (like thyroid function) as instrumental variables (IVs) [9]. This method inherently minimizes traditional confounding and is less prone to reverse causation bias, offering stronger causal inference than observational designs. MR has already been employed to investigate potential causal risk factors for MD, such as circulating cytokine levels [10]. However, the causal relationship between thyroid function—specifically TSH, FT4, and hyperthyroidism—and MD has not been established.

In this study, we hypothesize that thyroid dysfunction, particularly hyperthyroidism, may serve as an independent risk factor for MD. It is important to emphasize that we evaluate the causal effects of genetically predicted lifelong differences in thyroid hormone levels and thyroid disease liability, which represent a different physiological and clinical context from acutely manifest thyroid dysfunction or treatment‐related thyroid abnormalities. By leveraging MR, we aim to clarify the causal relationship between thyroid function and MD, providing insights that could address a critical gap in understanding MD pathophysiology and identify new avenues for clinical intervention.

## 2. Materials and Methods

### 2.1. Study Design

**FIGURE 1:**
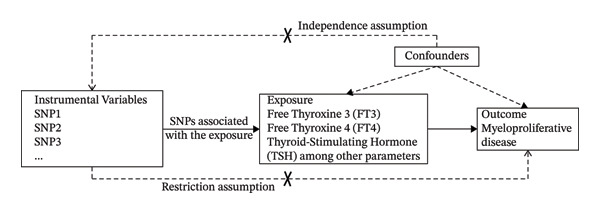
The flow diagram of the process in this Mendelian randomization analysis.

To investigate the causal relationship between thyroid function and MD, this study employed a two‐sample MR approach. The methodology adhered to the STROBE‐MR reporting checklist [11] and relied upon three core MR assumptions [12]: (1) the relevance assumption, requiring instrumental variables (SNPs) to be strongly associated with the exposure; (2) the independence assumption, positing that SNPs are independent of confounding factors; and (3) the exclusion restriction assumption, stating that SNPs influence the outcome exclusively through the exposure. Since this research used publicly available GWAS summary data, further ethical approval was not required. The analytical process is outlined in Figure 1.

### 2.2. Data Sources

This study utilized GWAS datasets to evaluate the causal relationship between thyroid function and MD. GWAS data for MD were obtained from the Finnish R12 cohort, which includes participants of European ancestry, utilizing two datasets: one for MD excluding chronic myelogenous leukemia (CML) and one for chronic MD (CMD). Exposure data for thyroid function traits, including thyrotropin (TSH), FT4, free triiodothyronine (FT3), total triiodothyronine (TT3), FT3/FT4 ratio, TT3/FT4 ratio, high TSH, and low TSH, were obtained from The ThyroidOmics Consortium. In addition, GWAS summary statistics for hyperthyroidism and hypothyroidism were obtained from the IEU Open GWAS database and the GWAS Catalog. To improve interpretability, we considered two phenotype definitions for thyroid disease: self‐reported diagnoses derived from UK Biobank noncancer illness codes and clinically defined diagnoses curated in the GWAS Catalog. The self‐reported UK Biobank phenotypes were “hyperthyroidism or thyrotoxicosis” (GWAS ID: ukb‐b‐20289) and “hypothyroidism or myxedema” (GWAS ID: ukb‐b‐19732). The clinically defined datasets were hyperthyroidism (GWAS Catalog ID: ebi‐a‐GCST90018860) and hypothyroidism (GWAS Catalog ID: ebi‐a‐GCST90018862). Dataset details are provided in Table S1.

### 2.3. IV Selection

To ensure the validity of the MR analysis, IVs were selected based on the following criteria [13]: (1) SNPs significantly associated with thyroid function traits (e.g., TSH, FT4, FT3, hyperthyroidism, and hypothyroidism) were included if they met a genome‐wide significance threshold of *p* < 5 × 10^−8^; (2) only SNPs with a minimum minor allele frequency (MAF) > 0.01 were retained to ensure sufficient variability in the genetic data; (3) linkage disequilibrium (LD) was addressed by excluding variants with an *R* ^2^ > 0.001 within a 10,000‐kb window [14]; (4) when selected SNPs were absent in the outcome GWAS datasets, proxy SNPs (with *R* ^2^ > 0.8) were identified and used as replacements [15]; (5) instrument strength was assessed using the F‐statistic, calculated as F = *R* ^2^ ∗ (N‐2) / (1‐*R* ^2^), where *R* ^2^ represents the proportion of variance explained by the SNP. SNPs with an F‐statistic > 10 were retained to minimize weak instrument bias [14].

### 2.4. MR Analyses

The primary analysis was conducted using the inverse‐variance weighted (IVW) method, which assumes no horizontal pleiotropy and provides a weighted average of individual SNP effects [16]. To address potential pleiotropy, additional sensitivity analyses were performed using complementary methods: (1) MR‐Egger regression: This method allows for the detection and correction of horizontal pleiotropy by estimating a nonzero intercept. (2) Weighted median estimator (WME): This approach provides a valid causal estimate even if up to 50% of the IVs are invalid. (3) Weighted mode: This method is robust when the largest cluster of SNPs represents valid instruments [17].

To further address possible statistical artifact, model instability, or weak instrument bias in the association between self‐reported hyperthyroidism/thyrotoxicosis and CMD, we applied several robust MR methods, including the Robust Adjusted Profile Score (MR‐RAPS) and penalized IVW analysis. These approaches help to reduce bias and improve the reliability of estimates, especially in the presence of weak instruments or outliers.

### 2.5. Sensitivity and Pleiotropy Analysis

To ensure the robustness of the results, several sensitivity analyses and pleiotropy tests were performed. Heterogeneity across SNPs was assessed using Cochran’s Q statistic. Horizontal pleiotropy was evaluated using MR‐Egger regression intercept and the Mendelian Randomization Pleiotropy RESidual Sum and Outlier (MR‐PRESSO) test. MR‐PRESSO also identified and corrected for outlier SNPs, refining causal estimates. Additionally, leave‐one‐out (LOO) analyses were conducted by iteratively excluding individual SNPs to assess their influence on the overall results [18]. Forest plots, scatter plots, and funnel plots were generated to visualize the results. All analyses were performed using R software (Version 4.0.5), incorporating the “TwoSampleMR” and “MR‐PRESSO” packages [19]. These tools provide a comprehensive framework for MR analysis and ensure reproducibility.

To further test the robustness and directionality of the observed associations, we performed the Steiger directionality test. This test statistically evaluates whether the genetic variants explain more variance in the exposure or the outcome, providing evidence that the causal direction is consistent with our hypothesis (i.e., that thyroid dysfunction influences CMD, rather than the reverse).

Additionally, to address the issue of extreme odds ratios (ORs) and wide confidence intervals, we conducted comprehensive outlier removal procedures. Outliers were identified using MR‐PRESSO, Radial MR, and LOO analyses. We iteratively removed outlying SNPs until all of the following conditions were met: MR‐PRESSO Global Test *p* > 0.05; MR‐Egger heterogeneity (Q‐test) *p* > 0.05; and MR‐Egger intercept (for horizontal pleiotropy) *p* > 0.05. We then re‐performed MR analyses on the cleaned dataset to improve the stability and reliability of the results. Furthermore, we applied false discovery rate (FDR) correction to adjust for multiple testing across all exposure–outcome pairs.

## 3. Results

To assess causal links between multiple thyroid function indicators and MDs (CMD and related conditions, excluding CML), we selected IVs for each indicator. The IV counts were as follows: 4 for TT3, 173 for TSH, 66 for FT4, 122 for hypothyroidism, 13 for hyperthyroidism, 15 for FT3/FT4 ratio, 8 for TT3/FT4 ratio, 29 for low TSH, and 6 for high TSH. Refer to Table S2–S3 for detailed IV screening information, proxy SNPs, and F‐statistics.

### 3.1. Reverse Causality Testing

To ensure the validity of the inferred causal direction, we performed Steiger directionality tests for all exposure–outcome pairs. As shown in Table 1, all positive associations in our MR analyses were supported by the Steiger tests (correct_causal_direction = TRUE), with significant *p* values (< 0.001), confirming that the genetic instruments explained more variance in the exposures (thyroid function traits) than in the outcomes (CMD or related diseases). This robustly supports the hypothesized direction of causality from thyroid function toward CMD and related phenotypes.

**TABLE 1:** Results of Steiger directionality test.

Exposure	Outcome	correct_causal_direction	steiger_pval
TT3/FT4 ratio	Chronic myeloproliferative disease	TRUE	5.6 E‐179
TT3/FT4 ratio	Myeloproliferative diseases (CML excluded)	TRUE	1.3 E‐174
Low TSH	Chronic myeloproliferative disease	TRUE	0
Low TSH	Myeloproliferative diseases (CML excluded)	TRUE	0
Autoimmune hyperthyroidism	Chronic myeloproliferative disease	TRUE	5.6 E‐111
Autoimmune hyperthyroidism	Myeloproliferative diseases (CML excluded)	TRUE	2.3 E‐113
Hyperthyroidism	Chronic myeloproliferative disease	TRUE	3.01 E‐70
Hyperthyroidism	Myeloproliferative diseases (CML excluded)	TRUE	6.83 E‐66
Hypothyroidism	Chronic myeloproliferative disease	TRUE	0
Hypothyroidism	Myeloproliferative diseases (CML excluded)	TRUE	0
FT3	Chronic myeloproliferative disease	TRUE	1.2 E‐102
FT3	Myeloproliferative diseases (CML excluded)	TRUE	5.9 E‐101
Hypothyroidism, strict autoimmune	Chronic myeloproliferative disease	TRUE	0
Hypothyroidism, strict autoimmune	Myeloproliferative diseases (CML excluded)	TRUE	0
TT3	Chronic myeloproliferative disease	TRUE	2.7 E‐177
TT3	Myeloproliferative diseases (CML excluded)	TRUE	6.9 E‐176
High TSH	Chronic myeloproliferative disease	TRUE	7.56 E‐39
High TSH	Myeloproliferative diseases (CML excluded)	TRUE	3.4 E‐30
FT3/FT4 ratio	Chronic myeloproliferative disease	TRUE	0
FT3/FT4 ratio	Myeloproliferative diseases (CML excluded)	TRUE	0
TSH	Chronic myeloproliferative disease	TRUE	0
TSH	Myeloproliferative diseases (CML excluded)	TRUE	0
FT4	Chronic myeloproliferative disease	TRUE	0
FT4	Myeloproliferative diseases (CML excluded)	TRUE	0
Noncancer illness code, self‐reported: hypothyroidism or myxedema	Chronic myeloproliferative disease	TRUE	0
Noncancer illness code, self‐reported: hypothyroidism or myxedema	Myeloproliferative diseases (CML excluded)	TRUE	0
Noncancer illness code, self‐reported: hyperthyroidism or thyrotoxicosis	Chronic myeloproliferative disease	TRUE	2.09 E‐55
Noncancer illness code, self‐reported: hyperthyroidism or thyrotoxicosis	Myeloproliferative diseases (CML excluded)	TRUE	2.2 E‐117

### 3.2. Outlier Removal and Sensitivity Analyses

We performed outlier detection using MR‐PRESSO, Radial MR, and LOO analyses, and re‐estimated causal effects after removing outlier variants identified by these methods. Full outlier SNP information is provided in Table S4.

### 3.3. MR Analysis Results

**FIGURE 2:**
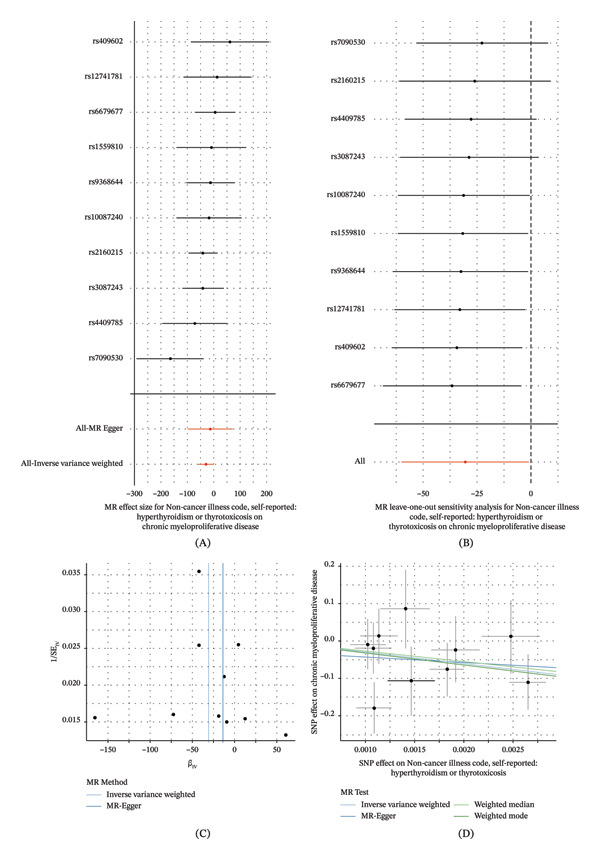
Association between self‐reported hyperthyroidism or thyrotoxicosis (UK Biobank noncancer illness code) and chronic myeloproliferative disease. Results are shown as (A) forest plot, (B) leave‐one‐out analysis, (C) scatter plot, and (D) funnel plot. Note: The extremely small odds ratio and unusually wide confidence interval in panel (A) suggest statistical instability and a potentially implausible estimate. This may reflect phenotype misclassification in self‐reported UK Biobank data and/or sparse case counts for some instrument variants. Results should, therefore, be interpreted with caution.

Genetic prediction results indicated a statistically significant association between self‐reported hyperthyroidism or thyrotoxicosis and CMD (OR = 5^∗^10^−14^, 95% CI: 7.8 × 10^−27^–0.3935, *p* = 0.0432). However, no other exposures, including TT3, TSH, FT4, hypothyroidism, or the FT3/FT4 ratio, showed significant associations with either CMD or MD (CML excluded) in the primary IVW analysis (all *p* > 0.05, Table S5, Table S6, and Figure 2). For hyperthyroidism, nominal associations were observed, but their interpretation depended on phenotype definition and outcome definition. The UK Biobank self‐reported “hyperthyroidism or thyrotoxicosis” phenotype yielded an implausible and statistically unstable effect estimate for CMD, and the detailed estimate is reported in Table S7.

Following outlier removal, MR analysis confirmed a positive causal association between hyperthyroidism and MDs (CML excluded): Hyperthyroidism ⟶ MDs (CML excluded): OR = 0.8806 (95% CI: 0.7954–0.9750), *p* = 0.0144, FDR = 0.2015 (Table 2). After FDR correction for multiple testing, none of the exposure–outcome associations met the statistical significance threshold (all FDR‐adjusted *p* > 0.05). Noncancer illness code, self‐reported: hyperthyroidism or thyrotoxicosis ⟶ CMD: OR = 5.53 × 10^−14^ (95% CI: 7.78 × 10^−27^–0.3935), *p* = 0.0432, FDR = 0.353. This extreme point estimate, characterized by a near‐zero OR and an exceptionally wide confidence interval, likely represents a statistical artifact due to sparse case–control distribution in the genetic instruments rather than a biological effect.

**TABLE 2:** Outlier removal analysis.

Exposure	Outcome	nSNP	Method	OR (95% CI)	*p* value	FDR
Hypothyroidism, strict autoimmune	Chronic myeloproliferative disease	174	Inverse‐variance weighted	1.045 (0.8923–1.2238)	0.5848	0.9054
Hypothyroidism, strict autoimmune	Myeloproliferative diseases (CML excluded)	173	Inverse‐variance weighted	1.0219 (0.9545–1.094)	0.5342	0.8425
Autoimmune hyperthyroidism	Chronic myeloproliferative disease	12	Inverse‐variance weighted	1.1082 (0.9235–1.3299)	0.2694	0.7865
Autoimmune hyperthyroidism	Myeloproliferative diseases (CML excluded)	12	Inverse‐variance weighted	0.9847 (0.9187–1.0554)	0.6632	0.844
TT3/FT4 ratio	Chronic myeloproliferative disease	8	Inverse‐variance weighted	1.6997 (0.2275–12.6965)	0.6051	0.9054
TT3/FT4 ratio	Myeloproliferative diseases (CML excluded)	6	Inverse‐variance weighted	2.4959 (0.689–9.0421)	0.1637	0.4724
FT3	Chronic myeloproliferative disease	7	Inverse‐variance weighted	0.7595 (0.2883–2.0008)	0.5778	0.9054
FT3	Myeloproliferative diseases (CML excluded)	7	Inverse‐variance weighted	0.9999 (0.6806–1.4691)	0.9998	0.9998
FT4	Chronic myeloproliferative disease	64	Inverse‐variance weighted	0.9982 (0.6148–1.6206)	0.9942	0.9942
FT4	Myeloproliferative diseases (CML excluded)	64	Inverse‐variance weighted	1.1739 (0.972–1.4178)	0.0959	0.4724
High TSH	Chronic myeloproliferative disease	5	Inverse‐variance weighted	1.1883 (0.5682–2.4852)	0.6467	0.9054
High TSH	Myeloproliferative diseases (CML excluded)	4	Inverse‐variance weighted	1.0592 (0.7702–1.4566)	0.7235	0.8441
Hyperthyroidism	Chronic myeloproliferative disease	11	Inverse‐variance weighted	1.0439 (0.8096–1.346)	0.7405	0.9425
Hyperthyroidism	Myeloproliferative diseases (CML excluded)	10	Inverse‐variance weighted	0.8806 (0.7954–0.975)	0.0144	0.2015
Hypothyroidism	Chronic myeloproliferative disease	70	Inverse‐variance weighted	1.2002 (0.9813–1.4679)	0.0756	0.353
Hypothyroidism	Myeloproliferative diseases (CML excluded)	68	Inverse‐variance weighted	1.0184 (0.941–1.1021)	0.6512	0.844
Low TSH	Chronic myeloproliferative disease	29	Inverse‐variance weighted	1.0766 (0.9414–1.2312)	0.2809	0.7865
Low TSH	Myeloproliferative diseases (CML excluded)	29	Inverse‐variance weighted	1.0054 (0.9482–1.0661)	0.8561	0.9219
FT3/FT4 ratio	Chronic myeloproliferative disease	15	Inverse‐variance weighted	0.9313 (0.0494–17.5617)	0.9621	0.9942
FT3/FT4 ratio	Myeloproliferative diseases (CML excluded)	15	Inverse‐variance weighted	0.5916 (0.1603–2.1828)	0.4307	0.8425
Noncancer illness code, self‐reported: hyperthyroidism or thyrotoxicosis	Chronic myeloproliferative disease	10	Inverse‐variance weighted	5.53E‐14 (7.78 E‐27–0.3935)	0.0432	0.353
Noncancer illness code, self‐reported: hyperthyroidism or thyrotoxicosis	Myeloproliferative diseases (CML excluded)	11	Inverse‐variance weighted	0.0448 (4.89 E‐06–411.166)	0.5048	0.8425
Noncancer illness code, self‐reported: hypothyroidism or myxedema	Chronic myeloproliferative disease	116	Inverse‐variance weighted	2.929 (0.0837–102.5133)	0.5536	0.9054
Noncancer illness code, self‐reported: hypothyroidism or myxedema	Myeloproliferative diseases (CML excluded)	111	Inverse‐variance weighted	3.1275 (0.6166–15.8635)	0.1687	0.4724
TSH	Chronic myeloproliferative disease	168	Inverse‐variance weighted	0.7607 (0.5711–1.0131)	0.0614	0.353
TSH	Myeloproliferative diseases (CML excluded)	165	Inverse‐variance weighted	0.9627 (0.8522–1.0876)	0.5416	0.8425
TT3	Chronic myeloproliferative disease	4	Inverse‐variance weighted	0.9907 (0.5856–1.6763)	0.9723	0.9942
TT3	Myeloproliferative diseases (CML excluded)	4	Inverse‐variance weighted	0.8442 (0.6844–1.0414)	0.1138	0.4724

However, after applying FDR correction for multiple testing, none of the primary associations remained statistically significant (all FDR‐adjusted *p* > 0.05). This suggests that while nominal associations were observed, these findings should be interpreted as suggestive and require further validation. A comprehensive summary of all exposure–outcome pairs, after outlier removal and sensitivity analyses, is provided in Table S7.

### 3.4. Sensitivity Analyses

To comprehensively assess the robustness of our MR findings, we conducted a series of sensitivity and pleiotropy analyses for all exposure–outcome pairs. For the majority of exposure–outcome pairs, there was no evidence of significant heterogeneity or horizontal pleiotropy. Specifically, most analyses yielded nonsignificant results for Cochran’s Q statistic (PHeterogeneity > 0.05) and the MR‐Egger regression intercept (PPleiotropy > 0.05), indicating the absence of substantial heterogeneity or directional pleiotropy among the IVs.

However, heterogeneity was detected in a subset of analyses. Notably, the association between the TT3/FT4 ratio and MDs (CML excluded) showed significant heterogeneity (*Q* = 20.01, *p* = 0.006) and evidence of directional pleiotropy (MR‐Egger intercept *p* = 0.035), as did a few other exposure–outcome pairs (Table S8). Furthermore, the MR‐PRESSO global test identified outlier variants in several analyses, including the TT3/FT4 ratio and high TSH.

After removing outlier SNPs as identified by MR‐PRESSO, the residual heterogeneity and pleiotropy were eliminated in the main analyses (Tables 3, 4, 5). For all primary exposure–outcome pairs after outlier removal, PHeterogeneity and PPleiotropy values exceeded 0.05, supporting the robustness and reliability of our causal estimates.

**TABLE 3:** MR‐PRESSO pleiotropy analysis.

Exposure	Outcome	Raw		Outlier corrected		Global P	Number of outliers	Distortion P
		OR (CI%)	*p*	OR (CI%)	*p*			
TT3/FT4 ratio	Chronic myeloproliferative disease	1.6997 (0.2733–10.5715)	0.587229438	NA	NA	0.651333333	NA	NA
TT3/FT4 ratio	Myeloproliferative diseases (CML excluded)	1.382 (0.353–5.4104)	0.65628655	2.4959 (0.689–9.0421)	0.222457954	0.034666667	2	0.511666667
FT3	Chronic myeloproliferative disease	0.7595 (0.4219–1.3672)	0.394397638	NA	NA	0.8955	NA	NA
FT3	Myeloproliferative diseases (CML excluded)	0.9999 (0.7086–1.411)	0.999757803	NA	NA	0.586333333	NA	NA
FT4	Chronic myeloproliferative disease	0.9884 (0.6099–1.6018)	0.962427537	NA	NA	0.088333333	NA	NA
FT4	Myeloproliferative diseases (CML excluded)	1.1707 (0.9715–1.4107)	0.10252612	NA	NA	0.134333333	NA	NA
High TSH	Chronic myeloproliferative disease	1.1883 (0.5771–2.447)	0.66405657	NA	NA	0.477833333	NA	NA
High TSH	Myeloproliferative diseases (CML excluded)	1.2864 (0.5301–3.1216)	0.601615759	1.0592 (0.7702–1.4566)	0.746934683	< 0.001	2	< 0.000166666666666667
Hyperthyroidism	Chronic myeloproliferative disease	1.065 (0.835–1.3583)	0.621822415	NA	NA	0.216333333	NA	NA
Hyperthyroidism	Myeloproliferative diseases (CML excluded)	0.925 (0.8297–1.0312)	0.187194769	NA	NA	0.077666667	NA	NA
Hypothyroidism	Chronic myeloproliferative disease	1.2092 (0.9895–1.4776)	0.06750408	NA	NA	0.063	NA	NA
Hypothyroidism	Myeloproliferative diseases (CML excluded)	1.0794 (0.9822–1.1861)	0.117184518	1.0316 (0.9584–1.1104)	0.410099356	< 0.001	2	0.078333333
Low TSH	Chronic myeloproliferative disease	1.0766 (0.9547–1.2141)	0.238725311	NA	NA	0.7795	NA	NA
Low TSH	Myeloproliferative diseases (CML excluded)	1.0054 (0.9482–1.0661)	0.857401119	NA	NA	0.219166667	NA	NA
FT3/FT4 ratio	Chronic myeloproliferative disease	0.9313 (0.0604–14.3516)	0.960051745	NA	NA	0.642333333	NA	NA
FT3/FT4 ratio	Myeloproliferative diseases (CML excluded)	0.5916 (0.1603–2.1828)	0.44379853	NA	NA	0.231333333	NA	NA
Noncancer illness code, self‐reported: hyperthyroidism or thyrotoxicosis	Chronic myeloproliferative disease	0 (0–0.082)	0.06152384	NA	NA	0.579833333	NA	NA
Noncancer illness code, self‐reported: hyperthyroidism or thyrotoxicosis	Myeloproliferative diseases (CML excluded)	0.0448 (0–411.166)	0.519886536	NA	NA	0.3585	NA	NA
Noncancer illness code, self‐reported: hypothyroidism or myxedema	Chronic myeloproliferative disease	1.6856 (0.0507–56.0063)	0.770713161	NA	NA	0.052666667	NA	NA
Noncancer illness code, self‐reported: hyperthyroidism or thyrotoxicosis	Myeloproliferative diseases (CML excluded)	1.8162 (0.2271–14.5272)	0.574818164	1.0189 (0.2376–4.3699)	0.979944155	< 0.001	2	0.0135
TSH	Chronic myeloproliferative disease	0.7477 (0.574–0.9738)	0.032432617	NA	NA	0.9015	NA	NA
TSH	Myeloproliferative diseases (CML excluded)	0.9741 (0.8498–1.1165)	0.706495334	0.9492 (0.8394–1.0734)	0.407256598	0.00017	1	0.766
TT3	Chronic myeloproliferative disease	0.9907 (0.5856–1.6763)	0.974504646	NA	NA	0.429	NA	NA
TT3	Myeloproliferative diseases (CML excluded)	0.8442 (0.6844–1.0414)	0.211988229	NA	NA	0.559333333	NA	NA
TT3/FT4 ratio	Myeloproliferative diseases (CML excluded)/after exclusion	2.4959 (0.689–9.0421)	0.222457954	NA (NA ‐ NA)	NA	0.336285714	NA	NA
High TSH	Myeloproliferative diseases (CML excluded)/after exclusion	1.0592 (0.7702–1.4566)	0.746934683	NA (NA ‐ NA)	NA	0.511	NA	NA
Hypothyroidism	Myeloproliferative diseases (CML excluded)/after exclusion	1.0316 (0.9584–1.1104)	0.410099356	NA (NA ‐ NA)	NA	0.438142857	NA	NA
Noncancer illness code, self‐reported: hypothyroidism or myxedema	Myeloproliferative diseases (CML excluded)/after exclusion	1.0189 (0.2376–4.3699)	0.979944155	NA (NA ‐ NA)	NA	0.031285714	NA	NA
TSH	Myeloproliferative diseases (CML excluded)/after exclusion	0.9492 (0.8394–1.0734)	0.407256598	NA (NA ‐ NA)	NA	0.045	NA	NA

**TABLE 4:** MR‐Egger regression (after outlier removal).

Exposure	Outcome	Heterogeneity		Pleiotropy	
		Q statistic (IVW)	*p* value	MR‐Egger intercept	*p* value
TT3/FT4 ratio	Myeloproliferative diseases (CML excluded)	6.7757	0.2379	0.0362	0.3743
High TSH	Myeloproliferative diseases (CML excluded)	3.0451	0.3847	−0.0482	0.5321
Hyperthyroidism	Myeloproliferative diseases (CML excluded)	11.2145	0.2613	0.0177	0.5347
Hypothyroidism	Myeloproliferative diseases (CML excluded)	78.7028	0.1552	−0.0027	0.7584
Noncancer illness code, self‐reported: hypothyroidism or myxedema	Myeloproliferative diseases (CML excluded)	133.2971	0.0648	−0.0104	0.2942
TSH	Myeloproliferative diseases (CML excluded)	187.5185	0.1007	−0.0008	0.8721

**TABLE 5:** MR‐PRESSO global tests (after outlier removal).

Exposure	Outcome	Raw		Outlier corrected		Global test *p* value	Outlier	Distortion_P
		OR (95% CI)	*p*	OR (95% CI)	*p*			
TT3/FT4 ratio	Myeloproliferative diseases (CML excluded)	2.4959 (0.6890–9.0421)	0.2225	NA (NA–NA)	NA	0.3375		
High TSH	Myeloproliferative diseases (CML excluded)	1.0592 (0.7702–1.4566)	0.7469	NA (NA–NA)	NA	0.4885		
Hyperthyroidism	Myeloproliferative diseases (CML excluded)	0.8806 (0.7954–0.9750)	0.0369	NA (NA–NA)	NA	0.3485		
Hypothyroidism	Myeloproliferative diseases (CML excluded)	1.0184 (0.9410–1.1021)	0.6527	NA (NA–NA)	NA	0.167		
Noncancer illness code, self‐reported: hypothyroidism or myxedema	Myeloproliferative diseases (CML excluded)	3.1275 (0.6166–15.8635)	0.1715	NA (NA–NA)	NA	0.0645		
TSH	Myeloproliferative diseases (CML excluded)	0.9627 (0.8522–1.0876)	0.5424	NA (NA–NA)	NA	0.1035		

Overall, we found no robust evidence supporting causal effects of genetically predicted thyroid traits on CMD or MD excluding CML after correction for multiple testing. Nominal signals involving hyperthyroidism were phenotype‐dependent and did not withstand FDR correction.

## 4. Discussion

In this two‐sample MR study, we evaluated the potential causal effects of multiple thyroid–function traits on MD. Overall, most thyroid‐related exposures—including genetically predicted TSH, FT4, TT3, FT3, hypothyroidism, and hormone ratios—showed no clear evidence of a causal association with either CMD or MD excluding CML in the primary analyses. By contrast, signals involving hyperthyroidism were observed, but these signals differed by phenotype definition (self‐reported vs clinically defined) and by outcome definition (CMD vs MD excluding CML). Importantly, none of the hyperthyroidism‐related associations remained statistically significant after FDR correction, and they should be interpreted as hypothesis‐generating rather than confirmatory. Therefore, our findings should be interpreted as suggestive rather than definitive.

The nominal evidence linking thyroid dysfunction to myeloproliferative conditions is consistent with prior observational reports that thyroid abnormalities may co‐occur with hematologic disorders. For instance, thyroid dysfunction has been reported to be more prevalent in patients with thrombocythemia [8]. Thyroid abnormalities have also been described in hematologic malignancies such as chronic myeloid leukemia and Philadelphia‐negative MD, particularly in patients receiving interferon‐alpha or tyrosine kinase inhibitors (TKIs) [20, 21]. Because TKIs can affect thyroid function and are associated with hypothyroidism and thyroid autoimmunity [22–24], many observational associations may be confounded by treatment exposure, disease severity, and clinical surveillance, limiting causal inference.

Compared with conventional observational designs, MR can reduce confounding and reverse causation by leveraging genetic instruments [9]. In our analyses, Steiger directionality tests supported the hypothesized direction from thyroid traits to MD phenotypes (correct_causal_direction = TRUE with very small *p* values), suggesting that reverse causation is unlikely to explain the observed nominal associations. Nevertheless, directionality alone does not guarantee validity: differences in phenotype definitions, instrument strength, and pleiotropy remain important considerations, especially for complex traits such as thyroid disease and MD.

Importantly, our hyperthyroidism results need to be interpreted separately for CMD and for MD excluding CML, and separately for self‐reported and clinically defined exposures. First, using the UK Biobank self‐reported “hyperthyroidism or thyrotoxicosis” phenotype, we observed an extremely small OR for CMD (IVW OR = 5.53 × 10^−14^, 95% CI: 7.78 × 10^−27^–0.3935, *p* = 0.0432), which is biologically implausible in magnitude and suggests instability or artifact rather than a reliable effect estimate. Second, after outlier removal, the clinically defined hyperthyroidism analysis showed a nominal association with MD excluding CML (hyperthyroidism ⟶ MD excluding CML: OR = 0.8806, 95% CI: 0.7954–0.9750, *p* = 0.0144), but this association did not survive multiple‐testing correction (FDR = 0.2015). Because this association did not survive correction for multiple testing, we treat it as a provisional signal that requires independent replication. Notably, this OR = 0.8806 pertains to MD excluding CML, not CMD, and it indicates a protective (inverse) association rather than increased risk. The divergence between self‐reported and clinically defined phenotypes—and between CMD and MD excluding CML—highlights the likelihood of phenotype heterogeneity and emphasizes the need for replication in large, clinically validated datasets.

Several factors may explain why the direction and magnitude of the observed hyperthyroidism associations are difficult to reconcile with pathophysiologic expectations. Thyroid hormones can influence hematopoiesis and the bone marrow microenvironment by modulating hematopoietic stem/progenitor proliferation and differentiation [23, 25, 26], and hyperthyroid states have been linked to immune and inflammatory changes [5, 27]. Pathways central to myeloproliferative neoplasms, such as JAK–STAT signaling, may also interact with thyroid hormone status [28], and hyperthyroidism has been associated with increased inflammatory cytokine production [29]. These lines of evidence could plausibly support either direction of association depending on context, including disease subtype composition, surveillance intensity, treatment‐related thyroid dysfunction, and nonlinear hormonal effects. However, because our nominal hyperthyroidism signal for MD excluding CML was OR < 1, any mechanistic interpretation should be framed carefully as a potential inverse association (i.e., genetically predicted hyperthyroidism liability associated with lower MD risk), rather than asserting that hyperthyroidism “predisposes” individuals to disease. Alternatively, the inverse association could reflect residual bias from phenotype definition, selection, or pleiotropy that is not fully eliminated by sensitivity analyses.

The absence of robust associations for continuous thyroid function traits (TSH, FT4, TT3, FT3, and ratios) may suggest that genetically proxied variation within the physiological range does not materially influence MD risk, or that effects—if present—are nonlinear and not well captured by linear MR models [30]. Another possibility is that genetic instruments for circulating hormone levels capture regulatory set‐points rather than overt clinical thyroid dysfunction, whereas clinically manifest thyroid disease reflects additional environmental, autoimmune, and treatment‐related components that may be more relevant to hematologic phenotypes. Consistent with this, Lei et al. [31] previously proposed that extreme TSH levels might influence leukemia risk, yet we did not find convincing evidence supporting a causal role for TSH in CMD or MD excluding CML, which may reflect differences in outcome definition, population, or residual confounding in earlier observational work.

Methodologically, we attempted to strengthen inference through instrument‐strength assessment (minimum F‐statistic = 29.16), heterogeneity testing, MR‐Egger intercept evaluation, MR‐PRESSO outlier detection, LOO analyses, and additional robust MR methods (e.g., MR‐RAPS and penalized IVW). These analyses reduce—but cannot fully eliminate—concerns about pleiotropy, instability from small numbers of instruments (especially for hyperthyroidism), and phenotype misclassification. The extreme CMD estimate for self‐reported hyperthyroidism/thyrotoxicosis (OR 5.53 × 10^−14^) and very wide confidence intervals strongly suggest that the corresponding finding is not stable and may be driven by sparse case counts, separation, or measurement heterogeneity rather than a true biological effect.

Overall, the main contribution of this study is the absence of evidence for strong causal effects of common genetically predicted thyroid hormone traits on CMD and MD excluding CML. This is clinically and scientifically informative because it helps contextualize prior inconsistent observational findings and suggests that previously reported associations may be driven by nongenetic factors, clinical confounding, treatment exposure, or surveillance differences rather than large lifelong causal effects. Clinically, if future studies confirm these hypothesis‐generating signals and establish an association between thyroid dysfunction liability and MD risk, this could inform risk stratification and motivate closer hematologic monitoring in selected patients with thyroid disease [21, 22]. However, given that the only nominally significant clinically defined association was inverse (OR < 1) for MD excluding CML, the self‐reported CMD estimate was implausibly extreme, and all findings were attenuated after FDR correction, our results should be considered hypothesis‐generating and not ready for clinical translation. Independent replication using well‐phenotyped thyroid disease definitions and harmonized MD subtype outcomes will be essential before drawing firm conclusions [32, 33].

### 4.1. Limitations

This study has several important limitations. First, although all genetic instruments used in our analyses demonstrated strong F‐statistics (minimum *F* = 29.16), the number of available IVs for certain exposures—particularly hyperthyroidism—was limited, which may reduce the stability and precision of the causal estimates. Second, our findings are based exclusively on populations of European ancestry, restricting their generalizability to other ethnic groups. Given known differences in thyroid function genetics across populations, future studies should include more diverse cohorts. Third, the use of self‐reported diagnoses for hyperthyroidism and hypothyroidism in the UK Biobank introduces potential misclassification and heterogeneity in phenotype definitions, which could attenuate true associations or introduce bias. Notably, while we attempted validation using clinically defined thyroid dysfunction GWAS data, no significant associations were observed, underscoring the need for large‐scale, clinically verified datasets. Furthermore, the observed association between self‐reported hyperthyroidism or thyrotoxicosis and CMD was characterized by an extremely low OR (OR = 5 × 10^−14^, 95% CI: 7.8 × 10^−27^–0.3935), with a wide confidence interval. Despite extensive sensitivity analyses using robust MR methods—including penalized IVW and MR‐RAPS—the effect estimate remained implausibly extreme. This suggests the result may be affected by methodological limitations, such as limited instrument number and possible outcome misclassification, rather than reflecting a true biological effect. As such, the magnitude of this association should be interpreted with caution, and further replication in independent cohorts is warranted. Finally, the biological mechanisms linking hyperthyroidism and CMD remain incompletely understood and require further experimental and mechanistic investigation.

### 4.2. Future Directions

Future studies should aim to validate these findings in diverse populations and explore additional thyroid function markers, such as T3 and reverse T3, to capture a broader spectrum of thyroid dysfunction. Advanced approaches, such as single‐cell sequencing, could provide insights into the thyroid–CMD pathway by characterizing cell‐specific effects of thyroid hormones on hematopoietic stem cells and their microenvironment. Cross‐ethnic MR studies are also needed to assess the consistency of findings across populations. Prospective cohort studies and clinical trials could further elucidate the role of thyroid function in CMD progression and outcomes, paving the way for targeted prevention and treatment strategies.

## 5. Conclusion

In conclusion, our MR analysis does not provide evidence supporting a major causal role of genetically predicted thyroid function traits in the etiology of CMD or MD excluding CML after correction for multiple testing. The nominal associations involving hyperthyroidism did not remain significant after FDR correction and should be regarded as hypothesis‐generating. A key contribution of this work is that it helps rule out strong causal effects of common genetically proxied variation in thyroid function on MD risk, which may aid interpretation of prior observational reports. Further large‐scale studies with clinically adjudicated thyroid phenotypes and harmonized MD subtype definitions are required.

**Nomenclature**
MD: Myeloproliferative diseases
CMD: Chronic myeloproliferative disease
TSH: Thyroid‐stimulating hormone
FT4: Free thyroxine
MR: Mendelian randomization
GWAS: Genome‐wide association study
IVW: Inverse‐variance weighted
ET: Essential thrombocythemia
PV: Polycythemia vera
PMF: Primary myelofibrosis
CML: Chronic myelogenous leukemia
TKIs: Tyrosine kinase inhibitors

## Author Contributions

Shu Deng, Jun Yan, and Jianping Shen carried out the studies, participated in collecting data, and drafted the manuscript. Jingjing Xiang and Mengke Mao performed the statistical analysis and participated in its design. Zhiyin Zheng participated in the acquisition, analysis, or interpretation of data and drafted the manuscript.

## Funding

This study was supported by the Major Project Jointly Built by the Zhejiang Provincial Administration of Traditional Chinese Medicine and Zhejiang Province (GZY‐ZJ‐KJ‐24013), awarded to Shu Deng, and the “Leading Geese” Research and Development Plan of Zhejiang Province (2025C02199), awarded to Shu Deng.

## Disclosure

All authors read and approved the final manuscript.

## Ethics Statement

This article is a Mendelian randomization study. The data for this study were obtained from publicly available databases and published literature data and do not require ethical approval and written informed consent.

## Consent

Please see the Ethics Statement.

## Conflicts of Interest

The authors declare no conflicts of interest.

## Supporting Information

Additional supporting information can be found online in the Supporting Information section.

## Supporting information


**Supporting Information** Supporting Table S1—Detailed Information on GWAS Data. Supporting Table S2—Instrumental Variable Matching Process and Strength Assessment. Supporting Table S3—Comprehensive Instrument Variable (IV) Information. Supporting Table S4—Summary of Excluded SNPs and Reasons for Removal in Mendelian Randomization (MR) Analyses. Supporting Table S5—Mendelian Randomization Estimates for the Causal Effect of Thyroid Function Traits on Myeloproliferative Diseases Using the Inverse Variance Weighted (IVW) Method. Supporting Table S6—Mendelian Randomization (MR) Analysis Results. Supporting Table S7—Summary of Mendelian Randomization (MR) Estimates for the Causal Associations Between Exposures and Hematological Outcomes. Supporting Table S8—Sensitivity Analysis and Pleiotropy Analysis.

## Data Availability

All data generated or analyzed during this study are included in this article and supporting information files.
